# Identification and Characterization of a Novel Long Noncoding RNA that Regulates Osteogenesis in Diet-Induced Obesity Mice

**DOI:** 10.3389/fcell.2022.832460

**Published:** 2022-04-21

**Authors:** Zhekai Hu, Wei Qiu, Yuedi Yu, Xingwen Wu, Fuchun Fang, Xiaofang Zhu, Xiaoyang Xu, Qisheng Tu, Thomas E. Van Dyke, Elise F. Morgan, Jake Chen

**Affiliations:** ^1^ Division of Oral Biology, Tufts University School of Dental Medicine, Boston, MA, United States; ^2^ Department of Chemical and Materials Engineering, New Jersey Institute of Technology, Newark, NJ, United States; ^3^ Clinical and Translational Research, Oral Medicine, Infection, and Immunity, Harvard School of Dental Medicine, Forsyth Institute, Boston, MA, United States; ^4^ Department of Mechanical Engineering, Boston University, Boston, MA, United States; ^5^ Department of Developmental, Molecular and Chemical Biology, Tufts School of Medicine, Graduate School of Biomedical Sciences, Tufts University, Boston, MA, United States

**Keywords:** long noncoding RNAs, diabetic bone diseases, diet-induced obese, epigenetics, LncR-DBD, BMP4

## Abstract

As a precursor to type 2 diabetes mellitus (T2D), obesity adversely alters bone cell functions, causing decreased bone quality. Currently, the mechanisms leading to alterations in bone quality in obesity and subsequently T2D are largely unclear. Emerging evidence suggests that long noncoding RNAs (lncRNAs) participate in a vast repertoire of biological processes and play essential roles in gene expression and posttranscriptional processes. Mechanistically, the expression of lncRNAs is implicated in pathogenesis surrounding the aggregation or alleviation of human diseases. To investigate the functional link between specific lncRNA and obesity-associated poor bone quality and elucidate the molecular mechanisms underlying the interaction between the two, we first assessed the structure of the bones in a diet-induced obese (DIO) mouse model. We found that bone microarchitecture markedly deteriorated in the DIO mice, mainly because of aberrant remodeling in the bone structure. The results of *in vitro* mechanistic experiments supported these observations. We then screened mRNAs and lncRNAs from DIO bones and functionally identified a specific lncRNA, Gm15222. Further analyses demonstrated that Gm15222 promotes osteogenesis and inhibits the expression of adipogenesis-related genes in DIO *via* recruitment of lysine demethylases KDM6B and KDM4B, respectively. Through this epigenetic pathway, Gm15222 modulates histone methylation of osteogenic genes. In addition, Gm15222 showed a positive correlation with the expression of a neighboring gene, BMP4. Together, the results of this study identified and provided initial characterization of Gm15222 as a critical epigenetic modifier that regulates osteogenesis and has potential roles in targeting the pathophysiology of bone disease in obesity and potential T2D.

## Introduction

Obesity is known to be the main risk factor for type 2 diabetes. In concordance with the WHO, overweight and obesity account for 44% of the diabetes cases (Frühbeck et al., 2013). Type 2 diabetes mellitus (T2D) is most strongly associated with obesity, and the prevalence of obesity-related diabetes is expected to double to 300 million by 2025 (Dyson, 2010).

T2D is a non-insulin–dependent diabetes mellitus and a chronic metabolic disease that occurs due to an imbalance between insulin secretion and glucose metabolism (Brownlee, 2001). Patients with T2D have as much as 50%–80% higher incidence of bone disorders compared to those without T2D (Rubin and Patsch, 2016). Hyperglycemia adversely alters bone cell functions, causing decreased bone quality and delayed wound healing; diseases with this pathological mechanism are collectively referred to as diabetic bone disease (DBD) (Bouillon, 1991). DBD represents a severe health issue globally (Schwartz and Lane, 2018; Valderrábano and Linares, 2018). Ongoing research continues to examine the underlying mechanisms of DBD in hopes of optimizing patient treatment. However, the mechanisms and pathology of DBD are unclear and controversial. Characteristics of bone differ between type 1 diabetes mellitus (T1D) and T2D (Nilsson et al., 2017). Previous research indicates that patients with T2D have average or higher bone mineral density (BMD), suggesting that T2D primarily impacts bone quality rather than bone density (Osório, 2011; Napoli et al., 2017). Disrupted bone cell activity causes imbalanced bone metabolism, and the diabetic microenvironment facilitates epigenetic and transcriptional alteration (Vrtačnik et al., 2014; Sanches et al., 2017).

Recent developments in high-throughput sequencing have led to a renewed interest in long noncoding RNAs (lncRNAs) and shown their vital role in mediating the occurrence and pathogenesis of diseases, which challenges our previous understanding of protein function (Chen et al., 2016). lncRNAs are a family of transcripts with more than 200 nucleotides that do not translate into proteins. Emerging evidence suggests that lncRNAs participate in a vast repertoire of biological processes and play essential roles in gene expression and post-transcriptional processes (Kung et al., 2013). Mechanistically, lncRNA can influence the local transcription by *cis*- and *trans*-effect (Kung et al., 2013; Andersen et al., 2019). For example, the sequence of lncRNAs alters DNA’s local structure and then change expression of adjacent genes; lncRNAs recruit transcription regulatory factors or link the proximal elements into promotors for epigenetic modification (Engreitz et al., 2016). Thus, lncRNAs are implicated in aggregation or alleviation of human diseases.

Functionally, it is apparent that both lncRNAs and other cues such as DNA modifications are involved in the development and metabolism of bone tissue. Lysine methylation is one of the most characterized histone modifications (Shi, 2007). Osteoblasts have a histone H3 lysine 27 trimethylation (H3K27me3) landscape distinguishing them from the precursor cells. The induction of different histone modifications leads to increases in expression of bone morphogenic protein (BMP) and Homeobox (HOX) family genes (Ye et al., 2012). Moreover, previous studies have analyzed histone methylation associated with enzymes like the histone lysine demethylase (KDM) family. For example, activated lysine specific demethylase 6B (KDM6B) is implicated in the promotion of downstream genes (Hui et al., 2014). These studies highlight the importance of histone modifications and associated enzymes in bone metabolism. Characterization of the complex functions of epigenetic factors in osteoblast-mediated osteogenesis is necessary to develop potential interventions for bone diseases (Agger et al., 2008; Hui et al., 2014).

Moreover, previous studies demonstrated the correlation between lncRNA and the level of H3K27me3 in the promoter of target genes. For example, it was discovered that lncRNA Gm15055 recruits PRC2 to maintain H3K27me3 modification, which repressed the expression of HOXA (Liu et al., 2016). Other research has indicated that lncRNA Firre maintains the H3K27me3 modification by altering the structure of the X chromosome (Fang et al., 2020). Therefore, there is a clear linear relationship among lncRNA, histone modification, and target gene expression. However, studies of lncRNA and its role in histone modification in bone diseases are just emerging and require further analysis.

This study aims to establish the functional link between a particular lncRNA and bone quality changes induced by obesity and to elucidate the molecular mechanisms underlying this interaction. We discovered in a high-fat diet (HFD) prediabetic mouse model that obesity alters osteogenesis *via* mechanisms that involve lncRNAs. We have further identified and initially characterized a specific lncRNA, Gm15222, that promotes osteogenesis and might inhibit adipogenesis in obesity. Gm15222 can recruit histone lysine demethylase KDM6B and influence the methylation of osteogenic genes. On the basis of the results of this study, lncRNA Gm15222 appears to have strong potential in targeting bone pathophysiology in obesity and possibly T2D.

## Materials and Methods

### Animal Experiments

Mice used in this study were purchased from the Jackson Laboratory (JAX, Bar Harbor, ME, United States). In detail, 18-week-old male high-fat diet-induced obese (DIO) mice (12 weeks of HFD) (Stock Number: 380,050, Strain Name: C57BL/6J DIO, the Jackson Laboratory) and 18-week-old male ND mice (Stock Number: 000,664, Strain Name: C57BL/6J, the Jackson Laboratory) were used for microarray assay and comparison of gene characteristics. Nine-week-old male DIO mice (3 weeks of HFD) were generated *via* 3 weeks of HFD in 6-week-old wild-type (WT) mice (Stock Number: 000,664, Strain Name: C57BL/6J, the Jackson Laboratory), and 9-week-old male ND mice were generated via 3 weeks normal diet (ND) in 6-week-old WT mice (Stock Number: 000,664, Strain Name: C57BL/6J, the Jackson Laboratory). If not otherwise specified, mouse bone marrow mesenchymal stem cells (BMSCs) were obtained from 4- to 6-week-old male WT mice (Stock Number: 000,664, Strain Name: C57BL/6J, the Jackson Laboratory). All *in vivo* experiments in this study followed the Tufts University Institutional Animal Care and Use Committee guidelines.

### Micro-Computed Tomography

Femurs were obtained from 18-week-old DIO and ND mice and scanned by Bruker Skyscan micro-CT at 9-µm voxel resolution. One hundred and fifty µCT slices, corresponding to a 1.35-mm region distal to the growth plate, were acquired for analysis. Micro-CT data were processed using Ctan software (Bruker Skyscan, MA, United States) to quantify three-dimensional measures of bone microarchitecture, including trabecular number (Tb.N), trabecular separation (Tb.Sp), trabecular thickness (Tb.Th), and bone volume/tissue volume (BV/TV) (Bouxsein et al., 2010). All processes were conducted according to the manufacturer’s instructions.

### Histomicroscopical Analysis and Tartrate-Resistant Acid Phosphatase Staining

The femurs of DIO and ND mice were fixed in 4% paraformaldehyde, decalcified for 3 weeks, embedded with paraffin, and cut into 5-μm sections as described in our previous work (Wu et al., 2019). Tissue sections were prepared for hematoxylin and eosin (H&E) and Tartrate-resistant acid phosphatase (TRAP) staining as previously described. Images were acquired using an Olympus BX53 microscope (Olympus, Norfolk, VA, United States).

### Cell Culture

MC3T3-E1 cells were obtained from the American Type Culture Collection (Manassas, VA, United States) and cultured in modified essential medium (α-MEM, Gibco, Life Technologies, Carlsbad, CA, United States) with 10% fetal bovine serum (FBS; Gibco, Life Technologies, Carlsbad, CA, United States) and 1% (v/v) penicillin/streptomycin (Gibco, Life Technologies, Carlsbad, CA, United States). All cells were cultured in an incubator at 37°C with 5% CO_2_. Mouse BMSCs were isolated from 4- to 6-week-old male mice as previously described (Han et al., 2015). BMSCs used to investigate the long-period HFD were isolated from 18-week-old HFD and ND mice. BMSCs were maintained in Dulbecco’s modified Eagle medium (Gibco, Life Technologies, Carlsbad, CA, United States) with 10% FBS and 1% (v/v) penicillin/streptomycin. Osteogenic differentiation of MC3T3-E1 cells and BMSCs was achieved by using an osteogenic medium containing ascorbic acid (50 mg/ml; Sigma-Aldrich, St. Louis, MO, United States), 5 mM β-glycerophosphate (Sigma-Aldrich, St. Louis, MO, United States), and 10 nM dexamethasone (Sigma-Aldrich, St. Louis, MO, United States) for 4 days.

### siRNA Transfection

The Small interfering RNA (siRNA), or scrambled control, was designed and purchased from Thermo Fisher Scientific (Waltham, MA, United States). The transfection was processed using HiPerFect Transfection Reagent (Qiagen, Hilden, Germany) as described in the manufacturer’s guidelines. Briefly, cells were seeded at a density of 5 × 10^5^ cells per well in 12-well plates. After overnight incubation, the cells were transfected for 72 h with siRNAs targeting specific genes (final siRNA concentration of 10 nM) or scrambled control and then harvested for subsequent measurements.

### RNA Extraction, Reverse Transcription, and Real-Time Quantitative Polymerase Chain Reaction

Quick-RNA Miniprep Kit (ZYMO Research, Irvine, CA, United States) was used for total RNA extraction from cells. Total RNA of the femur was extracted using TRIzol reagent (Thermo Fisher Scientific, Waltham, MA, United States) as described in our previous study (Qiu et al., 2021). Moloney-Murine Leukemia Virus (M-MLV) reverse transcriptase was used for reverse transcription of 1 μg of total RNA (Thermo Fisher Scientific, Waltham, MA, United States) as described in the manufacturer’s instructions. Finally, PowerUP SYBR Green Master Mix (Thermo Fisher Scientific, Waltham, MA, United States) was used for qRT-PCR on a Bio-Rad iQ5 Thermal Cycler (Bio-Rad Laboratories, Hercules, CA, United States), and each sample was repeated with three technical replicates. Primers used for qRT-PCR are listed in Supplementary Table S1.

### Alkaline Phosphatase and Alizarin Red S Staining

One-Step™ NBT/BCIP plus Suppressor Substrate Solution (Cat. No. 34042, Thermo Scientific™, Waltham, MA, United States) was used to detect alkaline phosphatase (ALP) activity according to the protocol provided by Thermo Scientific™. Alizarin Red S (ARS; Cat. No. A5533, Sigma-Aldrich, St. Louis, MO, United States) was used for analyzing osteogenesis. ARS (40mM) was dissolved in 100 ml of Phosphate-buffered saline (PBS) with an adjusted pH of 4.2. Cells were washed with PBS and then fixed in 10% formalin for 5 min. After rinsing, the ARS solution was added for 10 min to perform ARS staining. dH_2_O was used for subsequent rinse. The ARS stain was then dissolved with 10% (v/v) cetylpyridinium chloride for further quantitation at 562-nm absorbance.

### Microarray Analysis

Femurs were collected from 18-week-old DIO and WT mice. Total RNA was extracted using TRIzol reagent as described in our previous work (Qiu et al., 2021). The quality of RNA was tested by RNA gel electrophoresis. Total RNA (5 μg) from each femur was sent to Arraystar Inc. (Rockville, MD, United States) for further microarray analysis. Arraystar Inc. also performed data extraction, analysis, and figure plotting.

### Western Blot

Total proteins were prepared using RIPA Lysis and Extraction Buffer (Thermo Fisher Scientific, Waltham, MA, United States) containing Halt™ protease and phosphatase Inhibitor Cocktail and EDTA (Thermo Fisher Scientific, Waltham, MA, United States). The concentration of total protein was quantified by the Pierce™ BCA Protein Assay Kit (Thermo Fisher Scientific, Waltham, MA, United States). The samples were separated by sodium dodecyl sulfate-polyacrylamide gel electrophoresis (SDS-PAGE) and transferred to a polyvinylidene difluoride membrane (Merck Millipore, Darmstadt, Germany). Membranes were blocked at room temperature with 5% skim milk for 1 h. The blots were incubated with primary antibodies against BMP2 (1:1,000, Cat. No. ab214821, Abcam, Waltham, MA, United States), BMP4 (1:1,000, Cat. No. 4680, Cell Signaling Technology, MA, United States), Runx2 (1:1,000, Cat. No. sc-390351, Santa Cruz, CA, United States), and β-actin (1:20,000, Cat. No. ab8226, Abcam, Waltham, MA, United States) overnight at 4°C. After being washed with Tris-buffered saline with Tween buffer (Cat. No. 20360, Thermo Fisher Scientific, Waltham, MA, United States), the blots were incubated with horseradish peroxidase (HRP)-conjugated anti-rabbit (1:10,000) or anti-mouse (1:10,000) at room temperature for 1 h. Finally, the protein bands were visualized using an ECL Chemiluminescent Substrate Reagent kit (Thermo Fisher Scientific, Waltham, MA, United States).

### Chromatin Immunoprecipitation

Chromatin immunoprecipitation (ChIP) was performed on MSCs (1 × 10^7^ cells per ChIP) using the EZ-Magna ChIP™ A/G Chromatin Immunoprecipitation Kit (Cat. No. 17-10086, Merck KGaA, Darmstadt, Germany) as described by the manufacturer. A 150-mm culture dish containing 20 ml of medium was used for cell culture. One percent of formaldehyde was used to fix cells; subsequently, cell lysis buffer was used to create appropriately sized chromatin fragments. Next, cross-linked DNA was sheared to 200–1,000 base pairs for further immunoprecipitation with KDM6B (1:50, Cat. No. ab38113, Abcam, MA, United States) and H3K27me3 (1:50, Cat. No. 9733, Cell Signaling Technology, MA, United States) antibody. Finally, qRT-PCR was used to analyze the immunoprecipitated DNA fragments. Primers used for qRT-PCR are listed in Supplementary Table S1.

### RNA Immunoprecipitation

RNA immunoprecipitation (RIP) was performed on MSCs (2 × 10^7^ cells/RIP) using the Magna RIP™ RNA-Binding Protein Immunoprecipitation Kit (Cat. No. 17-701, Merck KgaA, Darmstadt, Germany) as described by the manufacturer. A 150-mm culture dish containing 20 ml of medium was used for cell culture. One percent of formaldehyde was used to fix the cells; subsequently, cell lysis buffer was used to create appropriately sized chromatin fragments. Next, cross-linked DNA was sheared to 200-1,000 base pairs for further immunoprecipitation with KDM6B antibody (1:50, Cat. No. ab38113, Abcam, Waltham, MA, United States). Finally, reverse transcription and qRT-PCR were used to analyze the immunoprecipitated RNA fragments. Primers used for qRT-PCR are listed in Supplementary Table S1.

### Calvarial Bone Wound Model and Regeneration in Mice

Experimental calvarial defects, 2 mm in diameter, were created as we described in detail in our previous reports (Tu et al., 2007; Li et al., 2008). After anesthesia, two critical-sized calvarial defects with a diameter of 2 mm were created on both sides of the calvarial bone using a low-speed dental bur with saline rinse. A cylinder-shaped silk scaffold (SS) of 2 mm diameter and 2 mm thickness was gently placed into each defect using tissue forceps. Animals were randomly assigned into two groups of five defects each, receiving the following treatments: 1) SS seeded with osteoblasts (transfected with siRNA negative control); 2) SS seeded with siRNA-Gm15222–transfected osteoblasts. Cells were concentrated to 1 × 10^7^ cells/ml in medium and then seeded onto the SS by pipetting the cell suspension onto the materials. The cell/SS construct was incubated for an additional 4 h to enable cell attachment *ex vivo* prior to implantation. Mice were sacrificed at 4 weeks post-surgery for determination of wound healing, and femur samples including wound sites were harvested.

### Statistical Analysis

Data analysis and graph generation were performed using GraphPad Prism software. Two-tailed student’s t-tests examined differences between two groups. A one-way analysis of variance (ANOVA) was used in conjunction with Tukey’s multiple comparisons test to examine differences between multiple groups. *p* ≤ 0.05 was considered significant.

### Microarray Data Accession

Microarray data have been deposited to the Gene Expression Omnibus database with the identification number of GSE193922.

## Results

### DIO Mice Exhibit Pathological Changes in Bone Development, Formation, and Regeneration

**FIGURE 1:**
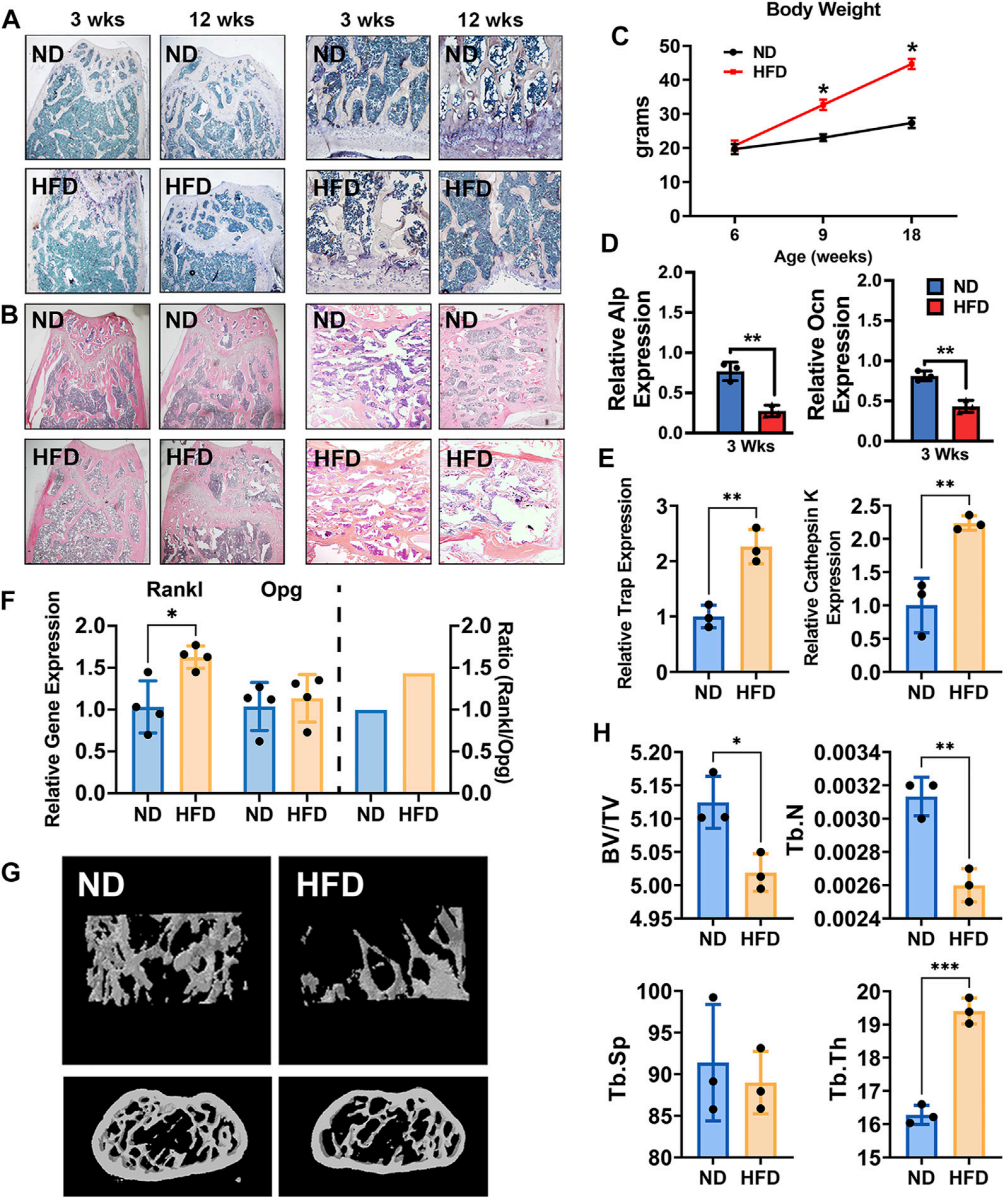
High-fat diet (HFD) leads to bone loss and alters bone metabolism in adult mice. **(A)** TRAP staining of femurs (left two panels) and vertebrae (right panels) of normal diet (ND) mice (18-week-old with ND) and HFD mice (18-week-old with 12 weeks of HFD). **(B)** H&E staining of femurs (left two panels) and vertebrae (right panels) of ND mice (18-week-old with ND) and HFD mice (18-week-old with 12 weeks of HFD) at 18 weeks of age. **(C)** The body weight curve. Male C57BL/6J DIO mice were fed HFD (60 kcal% fat) and control mice were fed normal diet (ND, 10 kcal% fat) between the ages of 6 and 18 weeks *n* = 4. Data were shown as mean ± S.D. **(D)** The mRNA expression of ALP (upper) and Ocn (lower), as detected by qRT-PCR in femurs of HFD and ND mice (3 weeks of HFD, *n* = 3). **(E)** The mRNA expression of TRAP and cathepsin K in femurs of HFD and ND mice (12 weeks of HFD, *n* = 3). **(F)** The mRNA expression of Opg and Rankl, as detected by qRT-PCR in femurs of HFD and ND mice (12 weeks of HFD, *n* = 4). **(G)** Representative images as detected and reconstructed by micro-CT and Ctan software in the femur of HFD and ND mice (12 weeks of HFD). **(H)** Histomorphometric analysis of micro-CT reconstruction of distal femurs, as measured by Ctan software, *n* = 3. Data were shown as mean ± S.D., two-tail *t*-test was used to test for those data between two groups. *p < 0.05; **p < 0.01; ***p < 0.001; ****p < 0.0001.

In this study, DIO mice were utilized as we and others described (Zhang et al., 2014; Yu et al., 2015; Fang et al., 2019; Wu et al., 2019; Wang et al., 2020; Harris et al., 2021; Qiu et al., 2021; Wu et al., 2022). These mice serve as an animal model of obesity and potential pre-T2D with elevated blood glucose and impaired glucose tolerance for a variety of metabolic studies including obesity, hyperglycemia, dyslipidemia, and glucose tolerance in T2D. Given that bone metabolism is a dynamic process in which gene expression varies during different developmental growth stages and HFD is a major risk factor for obesity that leads to T2D, we investigated the impact of the HFD on bone metabolism at various times, including 3 and 12 weeks. DIO mice exhibited pathological changes in bone development and formation. We observed bone metabolic changes in femurs including an increase in osteoclastogenesis. H&E staining showed morphological alterations after 3 and 12 weeks of HFD characterized by loosened spongious bone structures. Histomorphometric studies with TRAP staining also showed an increase in osteoclast activity in femurs and vertebrae at 3 and 12 weeks of HFD (Figures 1A,B). The bodyweight of the mice fed with HFD significantly increased compared to those fed with ND (Figure 1C).

Using primary cells, including bone marrow stromal cells (BMSCs) and bone marrow–derived macrophages (BMMs), an osteogenic environment was provided to BMSCs and osteoclast differentiation medium was provided to BMMs. ALP and OCN gene expression in BMSCs with osteogenic medium in both transient and short-period groups was significantly inhibited in DIO mice (Figure 1D). In contrast, osteoclast differentiation medium increased osteoclast-related genes, TRAP, and cathepsin K in BMMs (Figure 1E). Consistent with these findings, the ratio of the expression of RANKL to that of osteoprotegerin was increased to 1.5 in the femurs of DIO mice compared to the ND group, indicating increased osteoclastogenesis (Figure 1F). Evidence of imbalanced bone metabolism was also seen upon analysis of femoral bone microstructure by micro-CT. Femurs from the DIO mice showed lower BV/TV and Tb.N and higher Tb.Th, compared to ND (Figures 1G,H).

### Identification of a Specific lncRNA With Important Roles in Pathogenesis of Bone Disorder in Obese Mice

**FIGURE 2:**
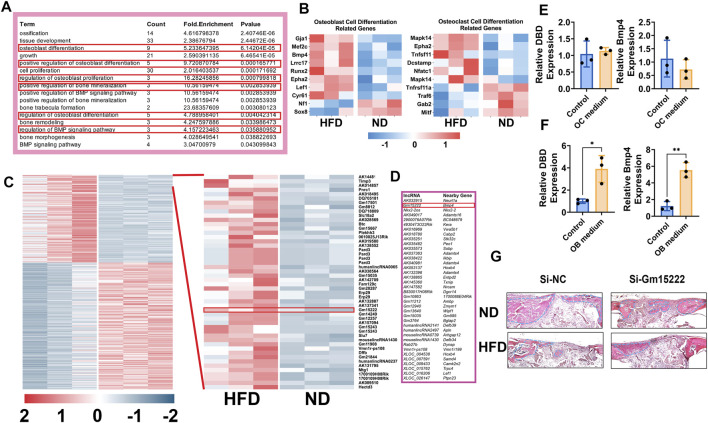
Identification of Gm15222 which has a distinct function in bone regeneration in the HFD mouse bones. **(A)** Upregulated genes annotated to a gene ontology (GO) term and example genes by gene ontology enrichment analysis of each component for ND mice and HFD mice. **(B)** Relative gene expression of osteogenesis (left) and osteoclastogenesis (right) markers ND and HFD mice. **(C)** The cluster heat map shows differentially expressed genes with expression change of more than two-fold from microarray data (three biological replicates per group, *p* < 0.05). Selected lncRNA has been highlighted in the red box. **(D)** lncRNAs classification and subgroup analysis showed the significantly expressed lncRNAs and their significantly expressed nearby genes. **(E)** BMMs were treated with or without osteoclasts differentiation medium [M-CSF (10 ng/ml) and RANKL (50 ng/ml)] for 5 days. The relative expression of Gm15222 and its related gene, Bmp4, were examined, *n* = 3. **(F)** BMSCs were treated with or without a mineralization medium [OM, ascorbic acid (50 μg/ml), 5 mM β-glycerophosphate] for 5 days, the relative expression of Gm15222 and its related gene Bmp4 were examined, *n* = 3. **(G)** Gm15222 deficiency inhibits bone regeneration in cranial bone of wild-type (WT) mice and high-fat-diet (HFD) mice. New bone was shown and highlighted with blue dotted lines. Data were shown as mean ± S.D., two-tail *t*-test was used to test for those data between two groups. *p < 0.05; **p < 0.01; ***p < 0.001; ****p < 0.0001.

All the above results indicate inhibition of osteogenesis and increased osteoclastogenesis in mice after short- and long-periods of HFD. We next sought to use RNA microarray to gain insight into the bone metabolic processes that produced these changes. Total RNA was extracted from bones of WT as well HFD mice with quality control. In the microarray assay, we observed differential expression of multiple genes including lncRNAs (305 upregulated lncRNAs, 401 downregulated lncRNAs, and 20,305 lncRNAs that were not differentially expressed) and mRNAs (205 upregulated mRNAs, 188 downregulated mRNAs, and 18,738 mRNAs that were not differentially expressed) (Volcano Plot, Supplementary Figure S1). We focused on bone formation-related genes and associated biological processes discovering that those molecules were significantly upregulated (Figures 2A,B). A lncRNA gene (Gm15222) was remarkably upregulated in DIO mice (Figure 2C). Gm15222 positively correlates with the evolution of BMP4 (Figure 2D), a potent osteogenic indicator that has been widely used in clinics for inducing bone regeneration and improving bone graft implants.

We hypothesized that lncRNA Gm15222 plays a role in bone metabolism in our DIO mouse model. To test this hypothesis, BMSCs and BMMs were cultured in osteoblastogenic and osteoclastogenic culture medium, respectively. Expression of lncRNA Gm15222 and BMP4 was unchanged during osteoclastogenesis in BMMs (Figure 2E), whereas the expression of lncRNA and BMP4 showed a significant increase when BMSCs were induced to osteogenic differentiation (Figure 2F). We next tested the function of Gm15222 in bone regeneration *in vivo*, using a cranial bone defect model. The osteoblasts transfected with si-Gm15222 were seeded onto SSs and placed into the cranial bone wound. After 4 weeks, bone regeneration was found to be retarded in DIO and ND mice in the si-Gm15222 group (Figure 2G).

### Expression of lncRNA Gm15222 Regulates the Expression of BMP4, Leading to a Downstream Impact on Osteo-Related Genes

**FIGURE 3:**
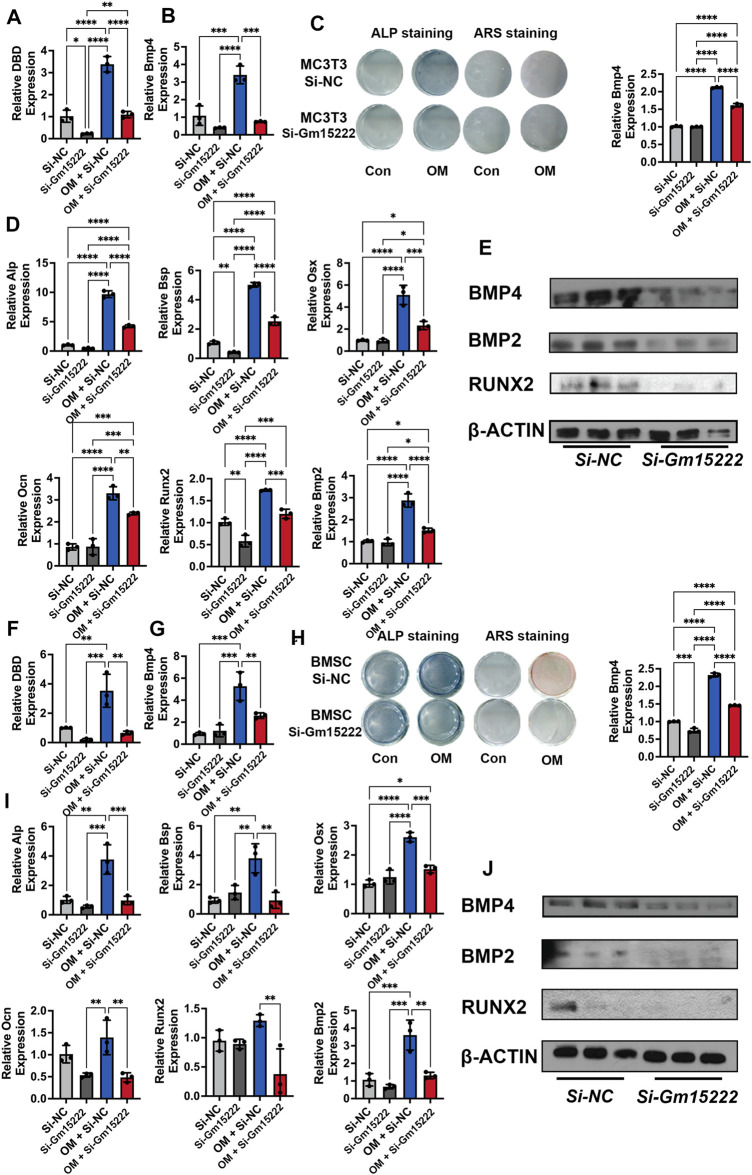
lncRNA Gm15222 upregulates the expression of Bmp4 and promotes osteoblast differentiation. **(A)** The interference efficacy of si-Gm15222, as detected by qRT-PCR in MC3T3-E1 cell line treated with or without mineralization medium, *n* = 3 [OM, ascorbic acid (50 μg/ml), 5 mM β-glycerophosphate]. Si-NC, siRNA negative control; Si-Gm15222, Gm15222 siRNA; OM, osteogenic medium. **(B)** The silence of Gm15222 inhibited its neighboring gene Bmp4 expression, as detected by qRT-PCR in MC3T3-E1 cell line treated with or without mineralization medium and si-Gm15222, *n* = 3. **(C)** The silence of Gm15222 impaired its neighboring gene Bmp4 expression as detected by ALP staining and Alizarin red R staining in MC3T3-E1 cell line treated with or without mineralization medium: the left two columns of plates were detected by alkaline phosphatase (ALP) staining; and the right two columns of plates were detected by Alizarin Red S (ARS) staining; the right column plot was the quantification of ARS staining, *n* = 3. **(D)** The silence of Gm15222 impaired osteogenic genes Alp, Bsp, Osx, Ocn, Runx2, and Bmp2 mRNA expression as detected by qRT-PCR in MC3T3-E1 cell line treated with or without mineralization medium and Si-Gm15222, *n* = 3. **(E)** The silence of Gm15222 impaired osteogenic genes Bmp4, Runx2, and Bmp2 protein expression as detected by Western blot in MC3T3-E1 cell line treated with or without Si-Gm15222, *n* = 3. **(F)** The interference efficacy of si-Gm15222 as detected by qRT-PCR in BMSCs cell line treated with or without mineralization medium and Si-Gm15222, *n* = 3. **(G)** The silence of Gm15222 impaired gene Bmp4 expression as detected by ALP staining and Alizarin red R staining in BMSCs cell line treated with or without mineralization medium and Si-Gm15222, *n* = 3. **(H)** The silence of Gm15222 inhibited the osteogenesis of BMSCs treated with or without mineralization medium and Si-GM15222: the left two columns of plates were detected by ALP staining, and the right two were detected by ARS staining; the right column plot was the quantification of ARS staining, *n* = 3. **(I)** The silence of Gm15222 impaired osteogenic genes Alp, Bsp, Osx, Ocn, Runx2, and Bmp2 expression, as detected by qRT-PCR in BMSCs cell line treated with or without mineralization medium, *n* = 3 [OM, ascorbic acid (50 μg/ml), 5 mM β-glycerophosphate] and Si-Gm15222. **(J)** The silence of Gm15222 impaired osteogenic Bmp4, Runx2, and Bmp2 protein expression, as detected by Western blot in BMSCs cell line treated with or without Si-Gm15222, *n* = 3. Data were shown as mean ± S.D., one-way ANOVA with Tukey’s *post hoc* test was used to test those data. *p < 0.05; **p < 0.01; ***p < 0.001; ****p < 0.0001.

To investigate the relationship between Gm15222 and its neighboring gene, BMP4, we downregulated the expression of Gm15222 in an osteoblastic cell line (MC3T3-E1) and BMSCs. The results revealed that the RNA and protein expression of BMP4 was positively correlated with the level of Gm15222. In MC3T3-E1 cells, the results showed that BMP4 was significantly decreased at both mRNA and protein levels when the expression of Gm15222 was inhibited (Figures 3A,B,E). Osteogenesis was subsequently determined by ALP and ARS staining revealing inhibition of osteogenic activity (Figure 3C). The expression levels of the osteogenic genes Alp, Bsp, Osx, Ocn, Bmp2, and Runx2 were decreased by the inhibition of Gm15222 as well (Figure 3D). The protein expression of BMP2 and RUNX2 also showed downregulation (Figure 3E). Similarly, the expression of BMP4 was downregulated while inhibiting Gm15222 in BMSCs (Figures 3F,G,J). Mineralization and osteogenic gene expression were reduced by inhibition of Gm15222 (Figures 3H–J)

### LncRNA Gm15222 Regulates the Expression of KDM Family Genes and Osteogenesis and Adipogenesis Distinctly

**FIGURE 4:**
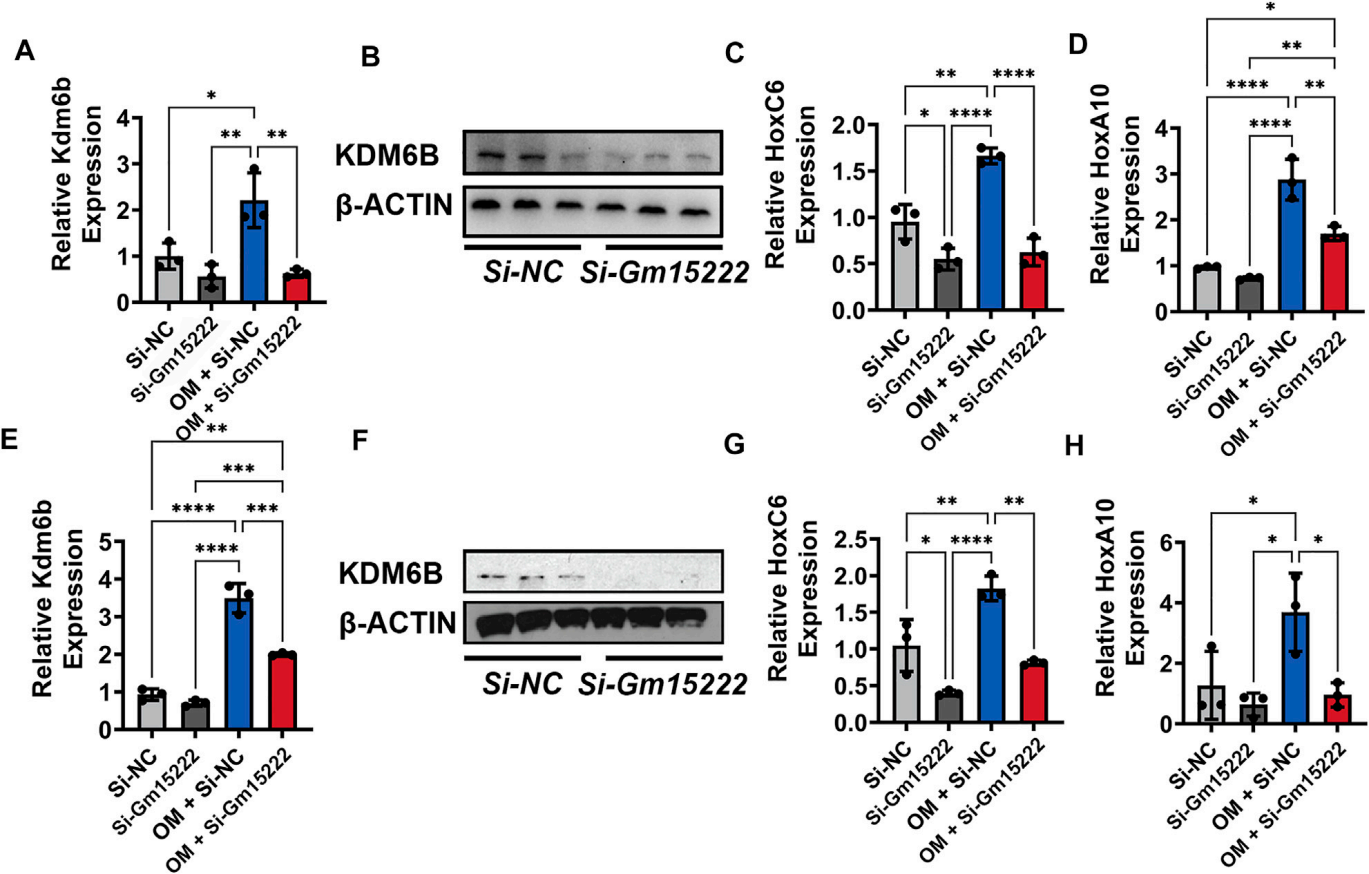
Gm15222 regulates the expression of Kdm6b and its downstream Hox gene family. **(A)** The silence of Gm15222 inhibited KDM6B expression, as detected by qRT-PCR in MC3T3-E1 cell line treated with or without mineralization medium and Si-Gm15222, *n* = 3. **(B)** The silence of Gm15222 impaired KDM6B protein expression in the process of mineralization, as detected by Western blot in MC3T3-E1 cell line treated with or without mineralization medium and Si-GM15222. **(C)** The silence of Gm15222 impaired HOXC6 expression, as detected by qRT-PCR in MC3T3-E1 cell line treated with or without mineralization medium and Si-Gm15222. **(D)** The silence of Gm15222 impaired HOXA10 gene expression, as detected by qRT-PCR in MC3T3-E1 cell line treated with or without mineralization medium and Si-Gm15222, *n* = 3. **(E)** The silence of Gm15222 inhibited KDM6B expression, as detected by qRT-PCR in BMSCs treated with or without mineralization medium and Si-GM15222, *n* = 3. **(F)** The silence of Gm15222 impaired KDM6B expression in the process of mineralization, as detected by Western blot in BMSCs treated with or without Si-Gm15222, *n* = 3. **(G)** The silence of Gm15222 impaired HOXC6 gene expression, as detected by qRT-PCR in BMSCs treated with or without mineralization medium and Si-Gm15222. **(H)** The silence of Gm15222 impaired HOXA10 expression, as detected by qRT-PCR in BMSCs treated with or without mineralization medium and Si-Gm15222, *n* = 3. Data were shown as mean ± S.D., one-way ANOVA with Tukey’s *post hoc* test was used to test those data. *p < 0.05; **p < 0.01; ***p < 0.001; ****p < 0.0001.

Histone demethylase KDM regulates osteogenesis and adipogenesis by reducing the level of methylation in the promotor of osteogenic genes and stimulating gene expression. To investigate the relationship between Gm15222 and KDM6B, we downregulated the expression of Gm15222 in MC3T3-E1 cells and BMSCs. We subsequently determined the expression level of KDM6B with qRT-PCR. The expression level of KDM6B was positively correlated with the Gm15222 expression level in both MC3T3-E1 cells (Figures 4A,B) and BMSCs (Figures 4E,F). It has been shown that the HOX gene is also stimulated by KDM6B (Ye et al., 2012). We determined the expression levels of the HOX family genes in this experiment. Results revealed that the expression of HOXC6 and HOXA10 decreased when Gm15222 was downregulated (Figures 4C,D,G,H). Given that HOX gene family expression is correlated with osteogenic genes, we might conclude that Gm15222 promotes osteogenesis.

Previous research has shown that adipogenesis and osteogenesis have an inverse relationship. As adipogenesis increases, osteogenesis is expected to decrease, and *vice versa*. We examined the impact of Gm15222 on adipogenesis as well. Downregulation of Gm15222 led to decreased expression levels of *KDM4B* and *DLX* genes (Suppl. Figures 2A,B), suggesting that Gm15222 might inhibit adipogenesis through the KDM4B-DLX-adipogenesis axis (Ye et al., 2012).

### LncRNA Gm15222 Recruits KDM6B and Induces Osteogenic Activity by Decreasing Methylation in the Promotors of BMP4 and HOXC6

**FIGURE 5:**
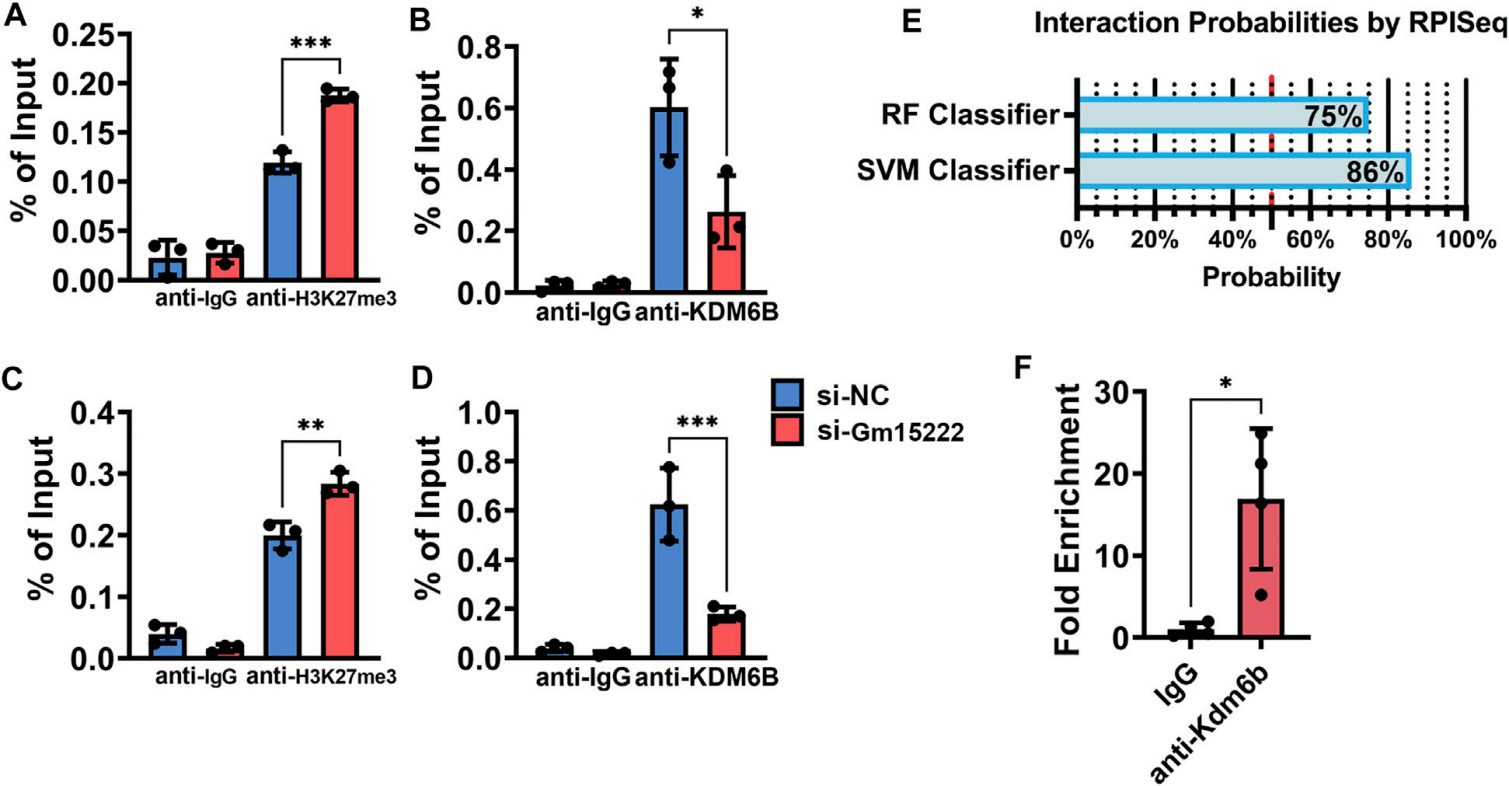
Gm15222 recruits KDM6B and alters the histone methylation in the promoters of BMP4 and HOXC6. **(A)** Quantification of BMP4 from retrieved Chromatin of BMSCs in the process of mineralization treated with or without si-Gm15222 in ChIP assay using H3K27me3 antibody, *n* = 3. SiNC, siRNA negative control. **(B)** Quantification of Bmp4 from retrieved Chromatin of BMSCs in the process of mineralization treated with or without si-Gm15222 in ChIP assay using Kdm6B antibody. **(C)** Quantification of HoxC6 from retrieved Chromatin of BMSCs in the process of mineralization treated with or without si-Gm15222 in ChIP assay using H3K27me3 antibody, *n* = 3. **(D)** Quantification of HoxC6 from retrieved DNAs of BMSCs in the process of mineralization treated with or without si-Gm15222 in ChIP assay using Kdm6B antibody, *n* = 3. **(E)** The interaction possibilities of Gm15222 and Kdm6b from RPISeq. **(F)** Quantification of Gm15222 from retrieved RNAs of BMSCs in RIP assay using Kdm6b antibody, *n* = 4. Data were shown as mean ± S.D., a two-tail *t*-test was used to test for those data between two groups, and a one-way ANOVA with Tukey’s *post hoc* test was used to test those data between more than two groups. *p < 0.05; **p < 0.01; ***p < 0.001; ****p < 0.0001.

Previous studies report that KDM6B transcriptionally activates BMP and HOX families, including BMP4 and HOXC6 (Ye et al., 2012). We speculated that there is a pathway that involves Gm15222, KDM6B, methylation, and osteogenesis. Therefore, we designed a ChIP experiment to examine changes in the level of histone methylation. Silencing of Gm15222 resulted in increased levels of histone methylation and decreased levels of KDM6B in the promotor of BMP4 and HOXC6 (Figures 5A–D), suggesting that less binding of KDM6B reduced the demethylation of the H3K27me3. We hypothesized that Gm15222 recruits KDM6B and forms an RNA-protein complex, inducing histone methylation in the promotor. We used RNA-Protein Interaction Prediction (RPISeq) to predict interaction probabilities. Results showed 86% interaction probability by SVM classifier and the 75% interaction probability by RF classifier (Figure 5E) (Muppirala et al., 2011). Subsequently, we used RIP to determine the existence of a complex. We found that the level of Gm15222 was around 10 times higher when using the KDM6B antibody than the control IgG (Figure 5F).

## Discussion

This study used a mouse DIO model to identify a novel lncRNA Gm15222. Consistent with our observations that a pre-T2D–relevant physiological state influences both osteogenesis and adipogenesis, we found that Gm15222 mediates methylation of osteogenic genes through recruitment of KDM6B. In addition, Gm15222 inhibits KDM4B and its downstream genes Dlx5 and Dlx6 suggesting the suppression of adipogenesis. On the basis of the results, we believe that we have discovered a new pathological mechanism for obesity-induced bone disease and other metabolic bone disorders.

In this study, DIO mice showed deterioration of bone microarchitecture with decreased BV/TV and Tb.N and increased Tb.Th values. The increased Tb.Th value may indicate a compensation in the long-term of HFD-associated DIO. The expression of osteoblast- and osteoclast-related genes positively correlates with disease, which is also consistent with previous studies. Diabetic bone disorder is an essential complication of metabolic alterations seen in obesity and T2D. Growing evidence indicates that individuals with T2D have impaired bone quality with diminished bone formation and increased marrow adiposity with a higher risk of fracture despite increased BMD (Valderrábano and Linares, 2018; Picke et al., 2019). In addition, a number of systemic reviews have demonstrated an increase in the risk of fragility fractures in patients with T2D (Janghorbani et al., 2007). In two animal models of T2D, DIO and KK-Ay, bone density is elevated with impaired trabecular architecture (Takagi et al., 2012). In addition to alteration in phenotype, bone-related genes and pathways contribute to dysregulation of the balance between osteoblastogenesis and osteoclastogenesis (Vrtačnik et al., 2014; Sassi et al., 2018). Therefore, abnormal bone metabolism with aberrant expression of genes in obesity may contribute to the dysregulation of bone formation and mineralization in diabetes.

The study of lncRNA in the skeletal system is an entirely novel area of research, and lncRNA activities are essential and requisite in pathophysiology. The functional mechanisms of lncRNAs vary; however, they can be divided into two main groups: *cis*-regulation and *trans*-transcriptional regulation (Yan et al., 2017). Several lncRNAs have been discovered that affect the pathogenesis of T2D: lncRNA betaFaar regulates islet beta-cell function and survival in T2D mice; lncRNA VEAL2 targets PRKCB2 contributing to reducing hyperpermeability in a hyperglycemic animal model (Sehgal et al., 2021; Zhang et al., 2021). This latter study suggests that lncRNA impacts the expression of genes that regulate bone mineralization. We found that Gm15222 controls DNA epigenetic modification in a DIO model. The expression of Gm15222 is positively correlated with that of BMP4, which is notably a key component of osteogenesis. Moreover, we present *in vitro* evidence showing that Gm15222 mediates the expression of downstream osteogenic genes. Overexpression of Gm15222 was shown to positively regulate osteogenesis and negatively regulate adipogenesis. Subsequent experiments demonstrated Gm15222-dependent recruitment of KDM6B demethylase and histone methylation levels of H3K27me3, which transcriptionally activated downstream genes. Various lncRNAs act as modular scaffolds or serve as recruiters of histone modification complexes (Engreitz et al., 2016). The results of the current study indicate that Gm15222 is involved in maintaining the compact chromatin structure of the BMP4 promoter through interactions with KDM6B. The downregulation of Gm15222 induced by siRNA inhibits the recruitment of Gm15222 to the BMP4 promoter region and then deactivates the transcription of BMP4. The BMP family (including BMP2, BMP4, and BMP7) is a potent inducer of osteogenic differentiation and can stimulate master transcription factors (Wang et al., 2014). In this study, we provide new insights into the contribution of BMPs to osteogenic differentiation. The silencing function of the H3K27me3 gene is critical for maintaining the homeostasis of differentiation in BMSCs. Hence, it is important to analyze the correlation between lncRNAs and histone modifications.

Osteoporosis is a typical bone metabolic disorder. It is related to a shift in the BMSC spectrum, where increased adipose tissue is accompanied by bone loss in the bone marrow compartment (Patel et al., 2015). In our study, we revealed a reduction of H3K27me3 in BMSCs with impaired Gm15222. These results suggest that Gm15222 may play an essential role relevant to obesity and diabetic bone loss by affecting the differentiation of BMSCs in the bone marrow. As a modifiable enzyme for histone demethylation, KDM6B can be activated or inactivated to determine the direction of BMSC-specific lineage differentiation (Huang et al., 2015). Gm15222 may serve as a mediator to recruit this modifiable enzyme to the correct site. We found that Gm15222 can recruit KDM6B, which selectively targets the BMP4 promoter region. Gm15222 promotes osteogenesis indirectly by enhancing the expression of its downstream molecule BMP4, which may provide feedback regulation with KDM6B. KDM6B stimulation during osteogenic differentiation can induce the expression of BMP4 by regulating levels of H3K27me3; however, upstream epigenetic mechanisms governing KDM6B-dependent BMP4 expression are unclear. By identifying Gm15222 as a positive regulator of BMP4, we determined the epigenetic regulation network of KDM6B-BMP4 signaling and osteogenic differentiation. Although this study demonstrated that Gm15222 regulates the chromatin state of the BMP4 promoter, the biological function of Gm15222 *in vivo* awaits further investigation. It is intriguing to consider that additional lncRNAs or chromatin modifiers might directly interact with Gm15222 to recruit it specifically to the BMP4 promoter region.

lncRNAs have emerged as critical regulators in multiple biological processes such as osteogenesis, osteoclastogenesis, and the immune response. lncRNAs play essential roles in the immune response, especially in macrophage polarization in the innate immune response (Wang and Zheng, 2018). Suryaji Patil and his colleagues reported that lncRNA directly or indirectly impacts bone metabolism and osteoporosis (Patil et al., 2020). The fact that Gm15222 is derived from DIO mouse tissues and our data indicating its critical role in epigenetic-related osteogenesis collectively suggest that lncRNA might impact the entire process of bone metabolism and thus have promise as a therapeutic target. This discovery has the potential to be utilized in regenerative medicine, especially in the treatment of human metabolic bone diseases, including obesity and diabetes-related bone disease.

**FIGURE 6:**
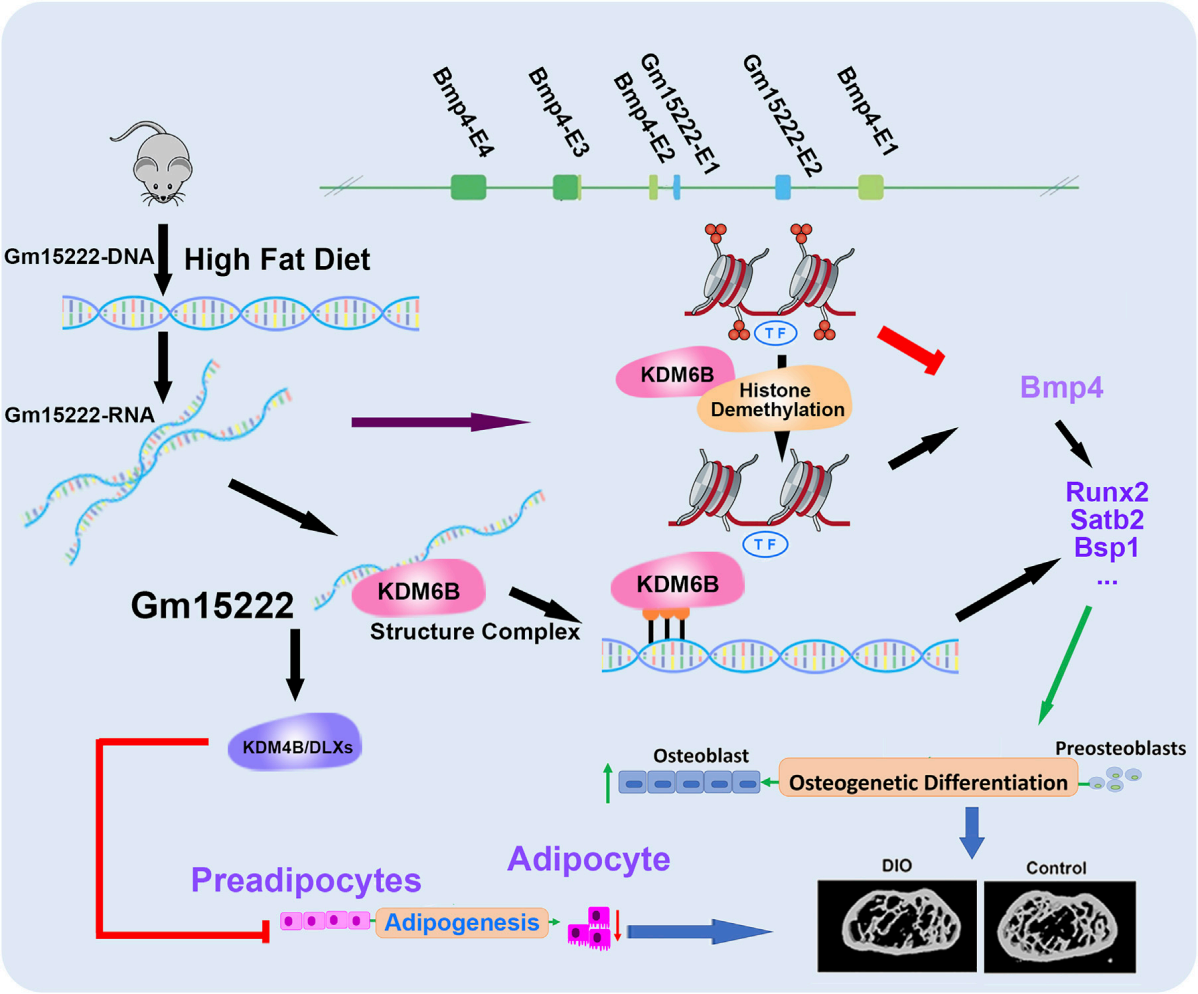
A schematic diagram showing our newly identified Gm15222 and the epigenetic mechanisms and potential of how Gm15222 targets the pathophysiology of diabetic bone disease (DBD). In detail, Bmp4 contains four exons (E1-E4), and Gm15222 contains two exons (E1-E2). lnR-Gm15222 can recruit KDM6B and reducing the modification of H3K27me3, thereby leading to the expression of osteogenic genes; meanwhile, to overcome the adipogenesis, Gm15222 might participate the KDM4B associated adipogenic inhibition.

In this study, we explored the regulatory mechanism between lncRNA and the epigenetic modulation in bone and found that KDM6B can be recruited by Gm15222 to target the modification of H3K27me3, leading to activation of osteogenesis. In our ongoing studies in this direction, we are applying more biological replicates for an extensive comparison between groups. Furthermore, we perform a set of independent metabolic phenotyping tests including HbA1c or blood glucose levels of the mice, instead of using the DIO mice directly purchased from the Jackson Laboratory. In summary, our research identifies lncRNA Gm15222 as a critical epigenetic modifier that regulates osteogenesis and adipogenesis (Figure 6). We provide new insights into the epigenetic modifications of bone metabolism and the metabolic regulation of bone tissue, and we suggest possible clues for the therapeutic intervention of pathological bone changes in obesity and possibly T2D.

## Data Availability

The datasets presented in this study can be found in online repositories. The names of the repository/repositories and accession number(s) can be found in the article/Supplementary Material.
