# Supplementary material for: Identification and Characterization of a Novel Long Noncoding RNA that Regulates Osteogenesis in Diet-Induced Obesity Mice
Source: Front Cell Dev Biol. 2022 Apr 21;10:832460. doi: 10.3389/fcell.2022.832460 (PMC9068931; doi:10.3389/fcell.2022.832460)
Supplement: Supplementary file 1 [file DataSheet1.pdf]

Identification and Characterization of a Novel Long Noncoding RNA that Regulates Osteogenesis in Diet-Induced Obesity Mice

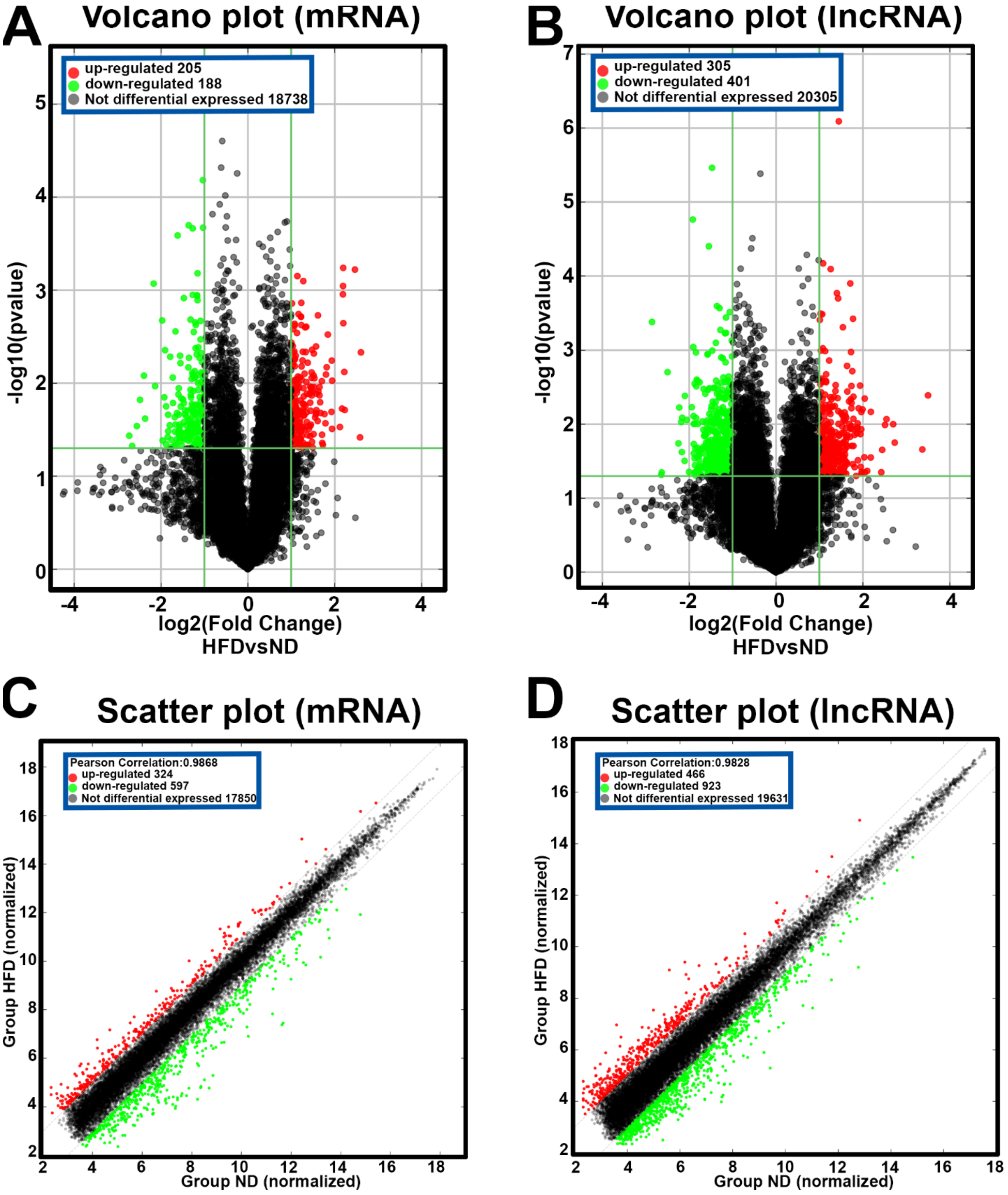

Suppl. Figure 1. High fat diet altered the mRNA and lncRNA expression in mouse bone. (A) The Volcano plot showed the different expressions of lncRNAs (A) and mRNAs (B) in the microarray assay. The Scatter plot showed the different expressions of lncRNAs (C) and mRNAs (D) in the microarray assay.

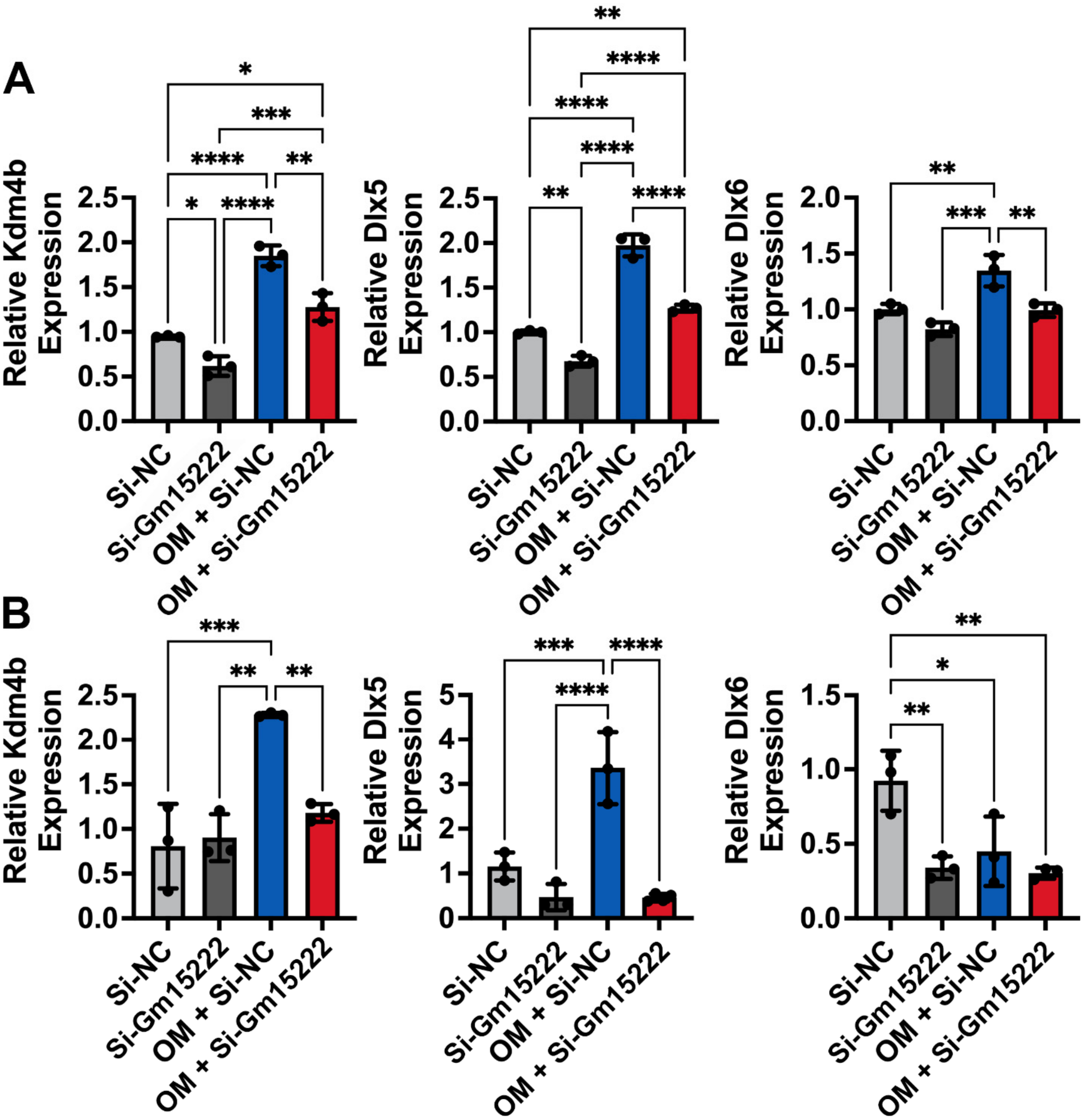

Suppl. Figure 2. (A) The silence of Gm15222 impaired KDM4B, DLX5, and DLX6 mRNA expression, as detected by qRT-PCR in MC3T3-E1 cell line, n=3. (B) The silence of Gm15222 impaired KDM4B, DLX5, and DLX6 mRNA expression, as detected by qRT-PCR in BMSCs, n=3. Data were shown as mean ± S.D., ANOVA test was used those data. *P < 0.05; ** P < 0.01; ***P < 0.001; ****P < 0.0001.

Supplemental Table 1. Primers for qRT-PCR and CHIP

m-DBD-F	TGGCGATATGCTAGCGACTG
m-DBD-R	GCCAAAGGCACAGGATTGTC
m-BMP4-F	TTCCTGGTAACCGAATGCTGA
m-BMP4-R	CCTGAATCTCGGCGACTTTTT
m-GAPDH-F	AGGTCGGTGTGAACGGATTTG
m-GAPDH-R	TGTAGACCATGTAGTTGAGGTCA
m-TRAP-F	CACTCCCACCCTGAGATTTGT
m-TRAP-R	CATCGTCTGCACGGTTCTG
m-C(TS)K-F	GAAGAAGACTCACCAGAAGCAG
m-C(TS)K-R	TCCAGGTTATGGGCAGAGATT
m-MMP9-F	CTGGACAGCCAGACACTAAAG
m-MMP9-R	CTCGCGGCAAGTCTTCAGAG
m-RANKL-F	CAGCATCGCTCTGTTCCTGTA
m-RANKL-R	CTGCGTTTTCATGGAGTCTCA
m-OPG-F	ACCCAGAAACTGGTCATCAGC
m-OPG-R	CTGCAATACACACACTCATCACT
m-ALP-F	CCAACTCTTTTGTGCCAGAGA
m-ALP-R	GGCTACATTGGTGTTGAGCTTTT
m-BMP2-F	GGGACCCGCTGTCTTCTAGT
m-BMP2-R	TCAACTCAAATTCGCTGAGGAC
m-OSX-F	ATGGCGTCCTCTCTGCTTG
m-OSX-R	TGAAAGGTCAGCGTATGGCTT
m-OCN-F	GCGCTCTGTCTCTCTGACCT
m-OCN-R	GCCGGAGTCTGTTCACTACC
m-RUNX2-F	CAGTCACCTCAGGCATGTCC
m-RUNX2-R	GTGCTGCTGGTCTGGAAGG
m-BSP-F	CAGGGAGGCAGTGACTCTTC
m-BSP-R	AGTGTGGAAAGTGTGGCGTT
m-KDM4B-F	AGGGACTTCAACAGATATGTGGC
m-KDM4B-R	GATGTCATCATACGTCTGCCG
m-KDM6B-F	TGAAGAACGTCAAGTCCATTGTG
m-KDM6B-R	TCCCGCTGTACCTGACAGT
m-HOXA10-F	CCTGCCGCGAACTCCTTTT
m-HOXA10-R	GGCGCTTCATTACGCTTGC
m-HOXC6-F	AATTCCACCGCCTATGATCCA
m-HOXC6-R	ACATTCTCCTGTGGCGAATAAAA
m-DLX5-F	TCTCTAGGACTGACGCAAACA
m-DLX5-R	GTTACACGCCATAGGGTCGC
m-DLX6-F	AAAACGACAGTGATCGAAAACGG

m-DLX6-R	AGTCTGCTGAAAGCGATGGTT
HoxC6_ChIP-F	ATAGCCCGACCAGGTAAAGG
HoxC6_ChIP-R	ATCATAGGCGGTGGAATTGA
BMP4_ChIP-F	TCCCGTGTCATGATGAAGTC
BMP4_ChIP-R	GCATGCACCCAGGGTAACTA
